# Supplementary material for: Safeguarding Bee Health: Insights from a Collaborative Monitoring and Prevention Project Against Pesticide Poisonings
Source: Animals (Basel). 2025 Feb 6;15(3):449. doi: 10.3390/ani15030449 (PMC11816341; doi:10.3390/ani15030449)
Supplement: Supplementary file 1 [file animals-15-00449-s001.zip › animals-3316322-supplementary.pdf]

Supplementary Table S1 - List of pesticides detectable with LC-MSMS and GC-MSMS analyses

List of pesticides detectable with LC-MS/MS and GC-MS/MS analyses			
**LC-MS/MS**			
Acephate	Dodine	Furathiocarb	Pirimicarb
Acetamiprid	Epoxiconazole	Hexaconazole	Pirimicarb-desmethyl
Aldicarb	Ethiofencarb	Hexythiazox	Pirimiphos-methyl
Aldicarb-sulfone	Ethion	Imazalil	Prochloraz
Aldicarb-sulfoxide	Ethirimol	Imidacloprid	Profenofos
Amitraz	Ethoprophos	Indoxacarb	Propamocarb
Azinphos-ethyl	Etofenprox	Iprovalicarb	Propaquizafop
Azinphos-methyl	Famoxadone	Isoprothiolane	Propargite
Azoxystrobin	Fenamidone	Isoxaflutole	Propham
Bitertanol	Fenamiphos	Kresoxim-methyl	Propiconazole
Bixafen	Fenamiphos-sulfone	Linuron	Propoxur
Boscalid	Fenamiphos-sulfoxide	Lufenuron	Prothioconazole-desthio
Bromuconazole	Fenarimol	Malaoxon	Pyraclostrobin
Bupirimate	Fenbuconazole	Malathion	Pyrazophos
Carbaryl	Fenhexamid	Mandipropamid	Pyrimethanil
Carbendazim	Fenoxycarb	Mefenpyr-diethyl	Quinoxyfen
Carbofuran	Fenpropathrin	Metalaxyl-M	Quizalofop-p-ethyl
Carbofuran-3-hydroxy	Fenpropidin	Metconazole	Spinosad
Chlorantraniliprole	Fenpropimorph	Methamidophos	Spirodiclofen
Chlorfenvinphos	Fenpyroximate	Methidathion	Spiroxamine
Chlorpropham	Fensulfothion	Methiocarb	Tebuconazole
Chlorpyriphos-ethyl	Fenthion	Methomyl	Tebufenozide
Clofentezine	Fenthion-oxon	Methoxyfenozide	Terbuthylazine
Clothianidin	Fenthion-oxon-sulfone	Metrafenone	Tetraconazole
Cyazofamid	Fenthion-oxon-sulfoxide	Metribuzin	Thiabendazole
Cyhalofop-Butyl	Fenthion-sulfone	Monocrotophos	Thiacloprid
Cymoxanil	Fenthion-sulfoxide	Myclobutanil	Thiametoxam
Cyproconazole	Flonicamid	N-(2,4-Dimethylphenyl)formamide	Thiencarbazone-methyl
Demeton-S-methyl	Flubendiamide	N-2,4-Dimethylphenyl-N`-methylformamidine	Thiodicarb
Demeton-S-methyl-sulfone	Flufenacet	Omethoate	Thiophanate-methyl
Demeton-S-methyl-sulfoxide	Flufenoxuron	Oxamyl	Tolclofos-methyl
Diazinon	Fluopicolide	Paclobutrazol	Triadimefon
Dichlorvos	Fluopyram	Parathion-ethyl	Triadimenol
Diethofencarb	Fluquinconazole	Penconazole	Triazophos
Difenoconazole	Flusilazole	Pencycuron	Trifloxystrobin
Dimethoate	Flutriafol	Phosalone	Triflumuron
Dimethomorph	Formetanate	Phosphamidon	Triticonazole
Diniconazole	Fosthiazate	Phoxim	Zoxamide
**GC-MS/MS**			
2-phenylphenol	Dicloran	beta-HCH	Permethrin
Acrinathrin	Dieldrin	gamma-HCH (Lindane)	Phenthoate
Aldrin	Diphenylamine	Heptachlor	Phosmet
Bifenthrin	Disulfoton	cis-Heptachlor-epoxide	Pirimiphos-ethyl
Biphenyl	Disulfoton-sulfone	trans-Heptachlor-epoxide	pp-DDD
Bromopropylate	alpha-Endosulfan	Iprodione	pp-DDE
Buprofezin	beta-Endosulfan	Isocarbophos	pp-DDT
Cadusafos	Endosulfan-sulfate	Isofenphos-methyl	Procymidone
cis-Chlordane	Endrin	Mepanipyrim	Propyzamide
oxy-Chlordane	EPN	Methacrifos	Pyridaben
trans-Chlordane	Etoxazole	Methoxychlor	Pyriproxyfen
Chlorfenapyr	Fenazaquin	Mevinphos	Spiromesifen
Chlorpyrifos-methyl	Fenitrothion	Nitrofen	Tebufenpyrad
Coumaphos	Fenvalerate	op-DDD	Tefluthrin
Cyfluthrin	Fipronil	op-DDE	Terbufos
lambda-Cyhalothrin	Fipronil-sulfone	op-DDT	Tetradifon
Cypermethrin	Fludioxonil	Oxadixyl	Tetramethrin
Cyprodinil	tau-Fluvalinate	Paraoxon-methyl	Trifluralin
Deltamethrin	Hexachlorobenzene	Parathion-methyl	Vinclozolin
Dicofol	alpha-HCH	Pendimethalin	
